# A Rare Case of GATA3 Positivity in Pleomorphic Lung Carcinoma in a Patient with History of Intracystic Papillary Carcinoma of the Breast: Primary Lung or Metastatic Disease?

**DOI:** 10.1155/2021/6664804

**Published:** 2021-01-20

**Authors:** Evi Abada

**Affiliations:** Department of Pathology, Wayne State University School of Medicine, 4707 St. Antoine Blvd. Detroit, Michigan 48201, USA

## Abstract

Pleomorphic lung carcinoma is a rare and aggressive neoplasm accounting for <1% of all lung tumors. It is more common in men and consists of spindle and/or giant cells with an epithelial component. In patients with known histories of malignancies at other sites, diagnosis of a new lung lesion may prove challenging with respect to classification as either primary or metastatic disease, especially in cases with overlapping immunohistochemical staining patterns. This was a case of a 67-year-old female with a newly discovered 1.5 cm nodule in her left lower lung lobe. Her past medical history was significant for an intracystic papillary carcinoma of the right breast diagnosed 8 years prior. Histopathologic examination of the new lung lesion revealed highly pleomorphic cells composed predominantly of neoplastic giant cells and atypical mitotic figures, with geographic areas of necrosis. However, no areas reminiscent of intracystic papillary carcinoma or other forms of breast carcinoma were seen. Immunohistochemistry showed that the tumor cells were immunoreactive for GATA3, TTF1, and napsin A and nonimmunoreactive for p40. Therefore, although this index lung tumor did show positivity with GATA3 staining, it was morphologically different from her original intracystic papillary carcinoma of the breast. In addition, intracystic papillary carcinomas are known to rarely metastasize to other organs, and GATA3 staining has been rarely reported in lung carcinomas. In summary, this case typifies the overlapping immunohistochemical staining patterns that may be seen in different tumors and the role of histopathologic morphology in arriving at the correct diagnosis.

## 1. Introduction

Pleomorphic lung carcinoma (PLC) is rare and accounts for 0.1 to 0.4% of all lung cancers [1]. Histologically, the World Health Organization classifies these tumors into 5 subtypes: pleomorphic carcinoma, spindle cell carcinoma, giant cell carcinoma, carcinosarcoma, and pulmonary blastoma [2]. PLC is defined as poorly differentiated non-small-cell lung carcinoma (NSCLC), namely, squamous cell carcinoma, adenocarcinoma, or large cell carcinoma, containing spindle cells and/or giant cells, or a carcinoma consisting of spindle and giant cells alone, with the pleomorphic component comprising at least 10% of the neoplasm [2, 3]. A diagnosis of PLC may be suspected on small biopsy or cytology but commonly requires a surgical resection to reach a conclusive definition [4]. The prognosis of PLC is worse than other forms of NSCLC and surgical resection, followed by appropriate adjuvant therapy might improve survival in these patients [5].

Intracystic papillary carcinoma (IPC) of the breast is a rare malignant tumor, constituting 1-2% of all breast carcinomas in women [6]. IPC often occurs in postmenopausal women. The typical characteristics are a mass with bloody nipple discharge and imaging findings of a round, oval, or lobulated circumscribed mass due to the appearance of a dilated duct, rather than the tumor within it [7]. IPC of the breast is an entity described in the 2019 World Heaalth Organization (WHO) classification of breast tumors and is considered to be a noninvasive carcinoma or a low-grade or indolent form of invasive carcinoma [8]. Histologically, the tumor is encysted within a dilated duct with arborization of the fibrovascular stroma and contains nodules of papillary carcinoma surrounded by a thick fibrous capsule [9]. Although IPC is considered a variant of ductal carcinoma in situ, some studies suggest that it may be metastatic [10–12], although this phenomenon is extremely rare. In clinical practice however, IPC is considered a low-risk invasive form of breast cancer.

GATA-binding protein 3 (GATA3) is a transcription factor of the GATA family and thus far has been used in surgical pathology as a marker for breast and urothelial carcinomas [13]. However, few studies have reported GATA3 positivity in about 2% of lung adenocarcinoma [14]. This was a case of a 67-year-old female with a past medical history of IPC and a newly discovered nodule in her left lower lung lobe. GATA3 positivity of the tumor cells raised the diagnostic possibility of a metastatic disease, versus a new lung primary.

## 2. Case Summary

**Figure 1:**
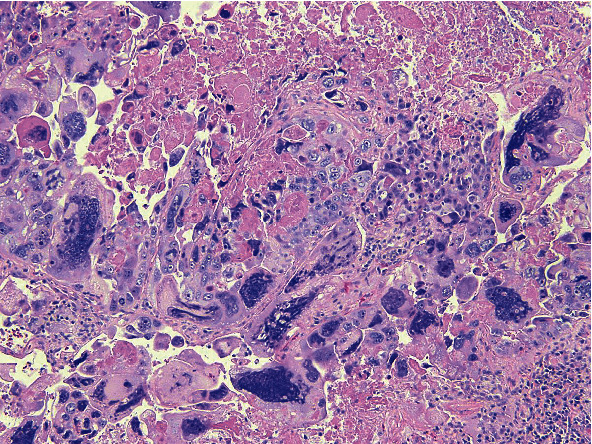
Hematoxylin and eosin stain and pleomorphic lung carcinoma (10x magnification).

**Figure 2:**
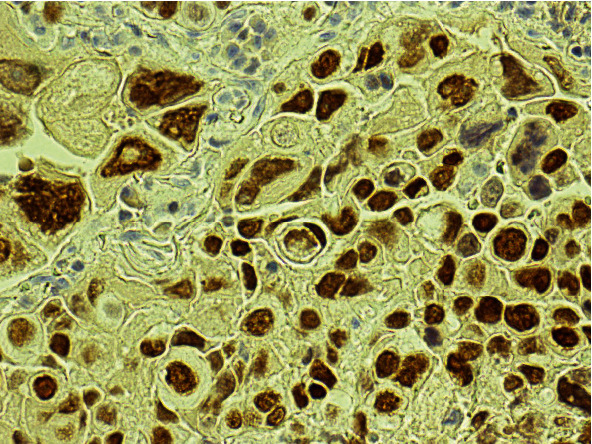
Tumor cells are strongly immunoreactive for GATA3.

**Figure 3:**
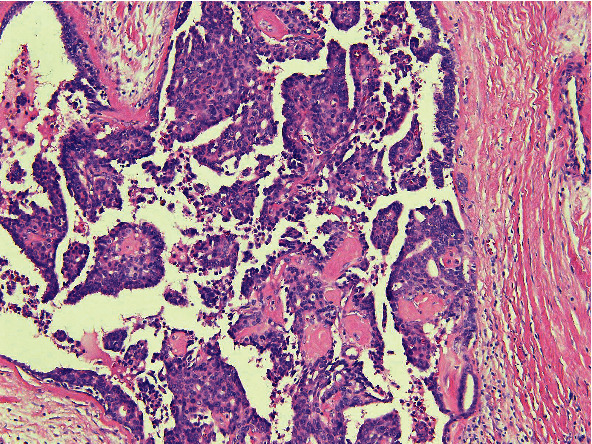
Hematoxylin and eosin stain and intracystic papillary carcinoma (10x magnification).

A 67-year-old female with past medical history significant for right-sided IPC of the breast, status post-lumpectomy, and radiotherapy, who was being managed with anastrozole presented with a new lung lesion discovered on a CT scan 8 years after her original breast cancer diagnosis. She was undergoing routine management for general malaise and decreased appetite. She had a significant past 40-year smoking history. Following an unsuccessful bronchial biopsy, a diagnostic thoracotomy with lobectomy was performed. On gross examination, a 1.5 × 1.2 × 1.1 cm well-defined tan-white solid mass was identified in the left lower lobe. A frozen section diagnosis of poorly differentiated carcinoma with extensive necrosis was made. Histopathologic examination of the permanent sections revealed highly pleomorphic cells composed predominantly of neoplastic giant cells and atypical mitotic figures, with geographic areas of necrosis, with a minor component of adenocarcinoma (Figure 1). However, no areas reminiscent of intracystic or other forms of breast carcinoma were seen. The tumor cells were strongly immunoreactive for GATA3 (Figure 2) and also showed positivity with TTF1 and napsin A. However, p40 was negative. Given the GATA3 immunoreactivity in the tumor cells, the possibility of metastatic breast carcinoma versus a primary lung carcinoma was raised. However, a comparison of the prior breast tumor (Figure 3) with the index lung lesion revealed classically distinct morphologies on histology. Furthermore, estrogen receptor (ER) and progesterone receptor (PR) which were both strongly positive in her breast tumor were both completely negative in the new lung mass. Therefore, the presence of a clearly identified TTF-1 positive adenocarcinoma component in the lung lesion and the low-grade nature of the patient's previous IPC warranted a diagnosis of a primary PLC.

## 3. Discussion

Pleomorphic lung carcinoma (PLC) is defined as poorly differentiated non-small-cell lung carcinoma (NSCLC), containing spindle cells and/or giant cells, or a carcinoma consisting of spindle and giant cells alone, with the pleomorphic component comprising at least 10% of the neoplasm [2, 3]. The morphology of the tumor in this case was consistent with a diagnosis of PLC because it mostly consisted of poorly differentiated cells with atypical mitotic figures, areas of geographic necrosis, and pleomorphic giant cells. The patient's 40-year smoking history is somewhat significant in this case as the majority of patients with PLC are known smokers with a large, peripheral mass characterized by well-defined margins [4]. Compared with other forms of lung cancers, PLC has a worse prognosis [15].

PLC may pose diagnostic problems, and immunohistochemistry is largely used when pathologists deal with these tumors in routine practice [4]. By immunohistochemistry, PLC tends to overexpress molecules associated with the epithelial-to-mesenchymal transition, such as vimentin, but a panel of immunostains including epithelial markers (cytokeratins, EMA), TTF-1, p40, and negative markers (e.g., melanocytic, mesothelial, and sarcoma-related primary antibodies) is helpful in the classification of this tumor [4]. The patient's tumor was positive for lung markers including TTF1 and napsin A, further supporting the consideration of a primary lung lesion, versus a metastatic disease.

The diagnostic surprise encountered in this case was when the patient's tumor cells were found to be strongly immunoreactive for GATA3. GATA3 has been thus far explored in surgical pathology as a marker for breast and urothelial carcinomas [13] and is frequently used to discriminate between primary or metastatic mammary and urothelial carcinoma, versus tumors originating from other organs. Therefore, in this patient with a known previous history of breast carcinoma and a new lung nodule, consideration of a metastatic disease versus a primary lung disease was entertained. However, histopathologic examination of the index lung lesion showed bizarre pleomorphic giant cells with a spindle cell component that was morphologically different from her intracystic breast carcinoma. In addition, whereas her IPC did show strong ER and PR positivity, ER and PR were both completely negative in the lung tumor. IPCs are known to be routinely strongly positive for ER and PR and are frequently human epidermal growth factor 2 (HER2) negative [16, 17], and that was exactly the experience with this patient's IPC.

Ultimately, based on the histopathologic examination and immunohistochemical results, a diagnosis of a primary PLC was made. Furthermore, a diagnosis of IPC was considered less likely because IPCs are known to rarely metastasize to other organs. In addition, GATA3 staining has been rarely reported in lung adenocarcinomas [13, 18]. In these studies, GATA3 was found to be positive in a limited number of primary lung adenocarcinomas. One other study reported the prognostic utility of GATA3 expression in patients with primary lung adenocarcinoma. High expression of GATA3 was found to be an independent risk factor for disease-free survival and overall survival in patients with primary lung adenocarcinomas [19].

## 4. Conclusion

GATA3 expression has been routinely used in surgical pathology to discriminate between tumors of breast and urothelial origins from other tumors. However, this case typifies the rare instance in which GATA3 may be positive in primary NSCLC. Therefore, additional studies are needed to further explore the diagnostic utility of GATA3 expression in primary lung tumors.

## Data Availability

No data were used to support this study.
